# A Randomized Clinical Study of the Plaque Removal Efficacy of a Novel Manual Toothbrush With Micro-Pulse Bristles on Fixed Orthodontic Patients

**DOI:** 10.7759/cureus.28453

**Published:** 2022-08-26

**Authors:** Abdulsalam Al Shammari, Fathima Fazrina Farook, Amal Fallatah, Sarah Aldosari, Khansa T Ababneh, Balsam M Aleissa

**Affiliations:** 1 College of Dentistry, King Saud Bin Abdulaziz University for Health Sciences, Riyadh, SAU; 2 Dental Hospital, Ministry of National Guard for Health Affairs, Riyadh, SAU; 3 Research and Development, King Abdullah International Medical Research Center, Riyadh, SAU; 4 Ministry of National Guard for Health Affairs, Dental Hospital, Riyadh, SAU; 5 Dental Hospital, Ministry of National Guard for Health Affairs,, Riyadh, SAU; 6 Ministry of National Guard For Health Affairs, Orthodontic Division, Dental Center, Riyadh, SAU

**Keywords:** toothbrush, prevention, oral health education, oral health, plaque control

## Abstract

Background

Fixed orthodontic appliances on tooth surfaces, such as brackets and bands, complicate oral hygiene and increase plaque accumulation, contributing to gingivitis, periodontitis, and tooth decay. While manual toothbrushes are an essential part of oral hygiene, there is little clinical evidence to demonstrate how effective manual toothbrushes with novel designs are at removing plaque from orthodontic patients. This study aims to evaluate three types of manual toothbrushes (Pulsar, conventional flat trim (C-TB), and orthodontic type (O-TB)) for their efficacy in plaque removal among patients undergoing fixed orthodontic treatment.

Methodology

The study followed the Consolidated Standards of Reporting Trials (CONSORT) guidelines. It was a three-treatment, three-period, examiner-blinded crossover clinical trial conducted with a single brushing exercise. Twenty-four subjects were randomized to one of three different bristle designs (Pulsar, C-TB, and O-TB). The primary outcome measure was the difference (baseline minus post-brushing) in plaque scores assessed using the Turesky-Modified Quigley-Hein Plaque Index during each study period.

Results

Of the 27 subjects enrolled in the study, 24 met the inclusion criteria and completed all three periods of the study. The mean age was 19.58 ± 1.55 years, with a range of 18-23 years. The differences between treatments in plaque score reduction after brushing were statistically significant (p-value <0.001). The treatment differences were statistically significant (p-value <0.001), favoring the C-TB toothbrush and the O-TB over the Pulsar design. On the contrary, the difference between the O-TB and C-TB types was not statistically significant.

Conclusions

C-TB and O-TB remove significantly more plaque than Pulsar toothbrushes after a single brushing exercise. Nevertheless, the C-TB tested in this study was more effective in removing dental plaque than the O-TB in patients wearing fixed orthodontic appliances. Considering the limitations of this study, additional research is required before evidence-based advice concerning the relative performance of the Pulsar toothbrushes in fixed orthodontic patients can be proven.

## Introduction

Dental biofilm contributes to the initiation and development of dental caries and periodontitis [1]. The important role that dental biofilm plays in gingivitis etiology has long been established and can be reversed through the removal of dental biofilm. To maintain optimal oral hygiene, it is crucial to effectively and efficiently control dental biofilm formation [2].

Mechanical plaque control with a regular toothbrush and fluoridated toothpaste plays a fundamental role in oral hygiene for primary prevention and is the most widespread means of controlling plaque at home and a primary preventive strategy for periodontitis [3-7]. Dental plaque accumulation, however, is influenced by several individual and material-related factors to prevent gingivitis, periodontitis, and decay [8]. One such factor is the fixed orthodontic appliance on tooth surfaces that creates difficulties in maintaining good oral hygiene due to the brackets, bands, archwires, and elastomeric modules that provide additional surface area for bacteria to develop and increase plaque retention [9,10]. In addition, the fixed orthodontic appliance has been found to be associated with changes in the type and composition of oral bacteria which combined with poor oral hygiene leads to favorable conditions for the development of periodontitis [11-13]. Twice daily toothbrushing with interproximal cleaning [14,15] is recommended as an essential part of a daily plaque control program for all orthodontic patients. Orthodontic patients face the challenge of maintaining good oral hygiene during treatment, as proper brushing techniques, interdental cleaning, and flossing techniques require more effort [16].

Several innovations have been made in the toothbrush head design aimed at improving the efficiency of the brush and overcoming non-ideal toothbrushing techniques and times [17-19]. Among the new developments in manual toothbrushes is the Oral-B Pulsar (Oral-B Pulsar Pro-Expert, Battery Powered Manual Toothbrush). It has a pressure-sensitive split head that adjusts to the contours of the teeth to clean surfaces with difficult access while maintaining a moderate amount of force on the teeth and gums. Moreover, the brush head features novel bristles (MicroPulse^TM^) that pivot independently (designed to penetrate deep between teeth) and a battery-powered gentle pulse action (designed to lift food and plaque) [20]. A few studies have reported a significant plaque removal ability of the Pulsar brush in non-orthodontic patients [20,21]. However, its effect on orthodontic patients has not been investigated.

Another innovation was a special toothbrush designed for orthodontic patients, the orthodontic toothbrush (O-TB) that featured a V-shaped groove on the long axis of the head, which is different from the conventional flat-trim brush (C-TB). It is hypothesized that the v-shaped bristles encourage better oral hygiene by increasing the contact between toothbrush bristles and orthodontic apparatus [15]. The O-TB is more effective in removing plaque than the C-TB, according to a recent systematic review and meta-analysis on the topic, but this improvement is not fully understood, underscoring the need for additional randomized clinical trials to evaluate the effects of using an O-TB in comparison to a C-TB [22].

It has been reported in the literature that powered particularly oscillating rotating brushes are superior to manual ones in non-orthodontic patients and reduced plaque and gingivitis more than manual toothbrushing in both the short and long term [23]. However, this is not true for patients wearing orthodontic appliances. A recent systematic review and meta-analysis concluded that there is no significant difference between powered and manual brushes in reducing plaque accumulation or gingivitis in patients with fixed orthodontic appliances [24]. There is limited evidence with conflicting reports regarding the effectiveness of advanced bristle designs of the manual toothbrush alone in removing plaque in patients undergoing orthodontic treatment [15,25-31].

The current study aims to clinically compare the effect of three types of manual toothbrushes (C-TB, O-TB, and the Pulsar) on plaque removal when the modified Bass method of brushing is used in patients with fixed orthodontic appliances.

## Materials and methods

**Figure 1:**
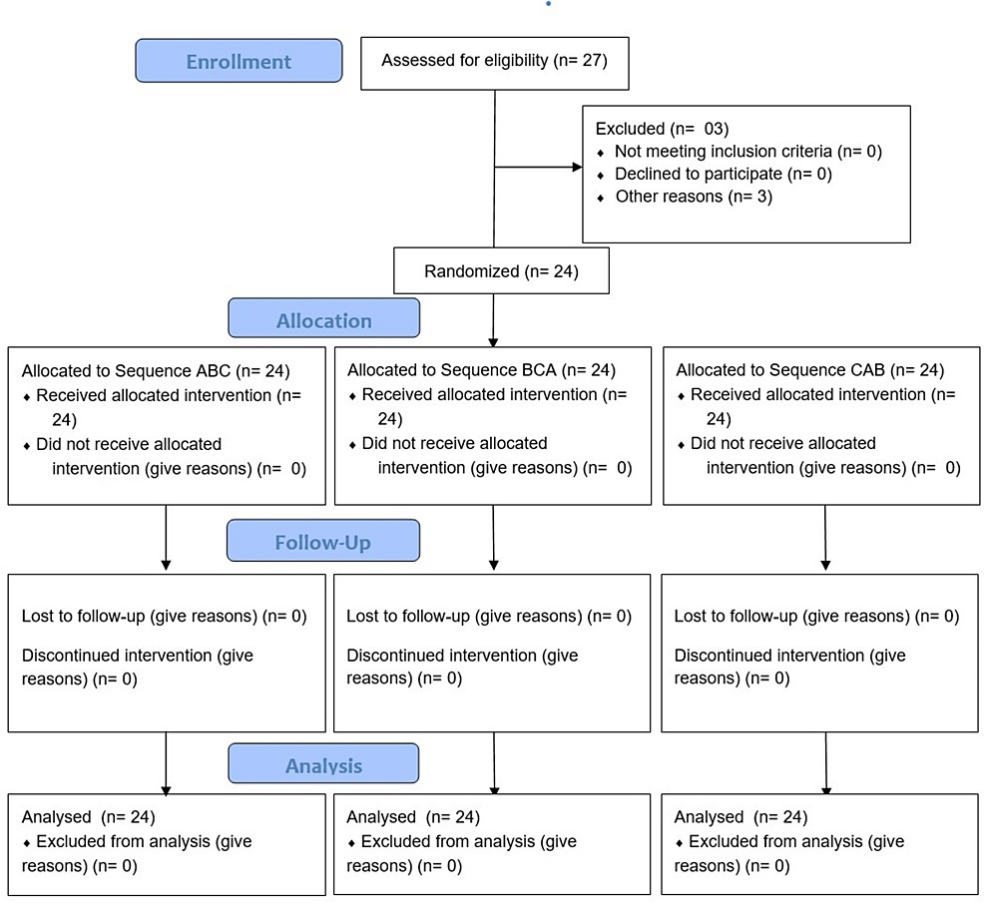
CONSORT flow diagram. CONSORT: Consolidated Standards of Reporting Trials

The experimental design was in accordance with the Consolidated Standards of Reporting Trials (Figure 1) [31]. In this 3 × 3, randomized, cross-over trial at the College of Dentistry, three manual toothbrush designs were compared in patients wearing fixed orthodontic appliances. Institutional Review Board approval was obtained for the study (RC20/085/R), which followed the Declaration of Helsinki. The study was conducted from November 2020 to June 2021 and was registered in the ISRCTN registry with trial ID ISRCTN15086076.

All participants between 18 and 25 years old, in good general and oral health, and wearing fully bonded fixed orthodontic appliances with a minimum of 25 natural teeth with facial and lingual scorable surfaces without any oral lesions or periodontal pockets of 3 mm or loss of attachment/recession of 2 mm were included in the study. The participants were also instructed to postpone elective dentistry, including prophylaxis, until the study was completed and to avoid participating in any other oral care studies while it was still ongoing.

The exclusion criteria were evidence of mucogingival problems, smoking, pregnancy, five or more carious lesions requiring restorative treatment, or wearing fixed or removable prosthesis. Participation in any other elective dental procedure, including prophylaxis, during the study period or the presence of any disease or condition that would interfere with the study procedures were additional grounds for exclusion.

The orthodontic residents approached all eligible patients attending the Orthodontic Clinic at the Dental Centre, Ministry of National Guard - Health Affairs, and gave them written study material. Only after getting their written informed consent were they included in the study.

Sample size estimation

The sample size was determined based on the previous plaque removal data from a similarly designed crossover study [31]. A sample of 24 participants assessed at three time points was used to achieve 80% power to detect a difference in the mean using a Geisser-Greenhouse Corrected F test at a 0.05 significance level. The standard deviation for the participants at the same time point was assumed to be 0.15. The pattern of the covariance matrix required all correlations equal with a correlation of 0.30 between the first and second time point measurements. The standard deviation of the hypothesized mean was 0.05. The calculation was performed in PASS software version 2020 [32].

**Figure 2:**
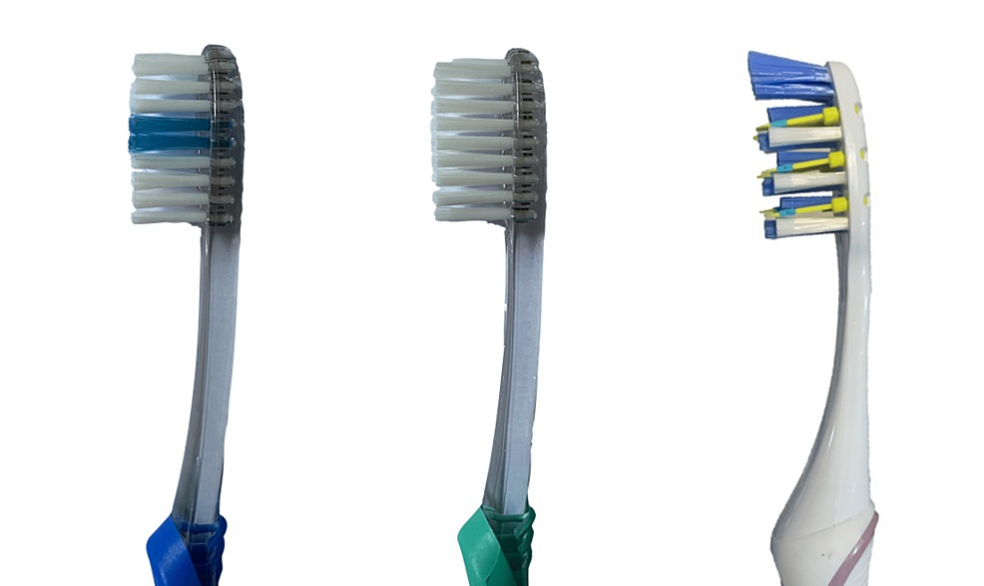
Three different bristle designs of commercially available Oral B manual toothbrushes. A. Orthodontic brush (Oral-B Pro-Expert Clinic Line Orthodontic Manual Toothbrush). B. Conventional Oral-B Pro Gum Care Manual Brush. C. Pulsar brush (Oral-B Pro-Expert Battery Powered Manual Toothbrush).

Three different bristle designs of commercially available toothbrushes (Pulsar, orthodontic, and flat trim) were used. The orthodontic brush used was the Oral-B Pro-Expert Clinic Line Orthodontic Manual Toothbrush, and the conventional brush was the Oral-B Pro Gum Care Manual Brush. The third Pulsar brush was an Oral-B Pro-Expert Battery Operated Manual Toothbrush. All brushes were adult brushes with soft brush heads (Figure 2).

Randomization (random number generation, allocation concealment, implementation)

The study subjects were randomly assigned to one of the three treatment sequences determined by a computer-generated plan prepared before the study (ABC, BCA, and CAB) with 24 participants in each treatment sequence. The distribution and allocation of toothbrushes were carried out by an investigator who was blind to the data collection process. To conceal allocations, sequentially numbered, opaque, sealed envelopes containing pre-prepared treatment allocation cards were used. The participants were recalled four times (visits 1, 2, 3, and 4) with a wash-out period of one week between each visit.

Participants were instructed not to brush or floss their teeth or use a mouth rinse for 24 hours before each appointment, as well as not to eat, drink, or smoke for four hours before each appointment. Two blinded evaluators evaluated and scored the plaque. The intraclass correlation (ICC) was used to calculate the interexaminer reliability for plaque scoring.

The patients were interviewed on day 1 and relevant information was gathered about their demography, oral hygiene habits, and dental clinical examination, including recording the number of teeth.

On the first day, all patients were given verbal and written instructions on how to utilize the assigned toothbrush with the modified Bass method. The toothbrush is positioned at the gum line at a 45° angle and is moved in short, vibratory strokes along the dental arches as well until all of the tooth surfaces that are accessible have been brushed. During each appointment, the patient used the designated toothbrush and standard fluoride toothpaste to brush their teeth for two minutes (30 seconds for each quadrant). After brushing, the participants were asked to rinse their mouths once lightly with water.

A red disclosing solution was used to disclose the plaque during each visit to gather baseline information. Plaque assessments were performed by two periodontists who were blinded to the treatment (type of brush) method using the Turesky Modified Quigley-Hein Plaque Index. The scores were re-evaluated by the same examiners independently after brushing and swishing with disclosing solution again. The examiners received prior training with the method.

The same procedure was repeated during subsequent appointments using the other toothbrushes that were randomly assigned to each participant. The same procedure was followed for all three periods. In Period 3, the participant accountability was documented.

Table 1 lists the criteria for plaque scoring using the Turesky Modified Quigley-Hein Plaque Index. The buccal and lingual surfaces of each tooth were scored; a total of 40 surfaces on 20 teeth were scored per participant. The average values for each participant were calculated by dividing the total score by the number of teeth examined.

**Table 1:** Turesky Modified Quigley-Hein Plaque Index.

Score	Criteria for plaque scoring
0	No plaque
1	Separate flecks of plaque present at the cervical margin
2	A continuous band of plaque up to 1 mm at the cervical margin
3	A band of plaque that is wider than 1 mm but covering less than one-third of the side of the crown of the tooth
4	Plaque covering at least one-third but less than two-thirds of the side of the crown
5	Plaque covering more than two-thirds of the side of the crown

Outcomes (primary and secondary) and any changes after trial commencement

The plaque scores were averaged for each participant to obtain a single whole-mouth average score at baseline and another whole-mouth average score following brushing. The outcome was the difference (baseline minus post-brushing) in the average scores calculated for each participant during each treatment period.

Throughout the study, all serious adverse events (AEs) were included in appropriate case reports, and all self-reported non-serious oral AEs were also recorded. Whole-body AE was recorded only if it could be related to the use of the device.

Blinding

Blinding of the patients was not possible. Plaque assessments were performed by two periodontists who were blinded to the treatment groups.

Statistical analyses

Descriptive statistics were used to summarize the sample characteristics and baseline dental measurements. Means and standard deviations (SD) were calculated for the three groups at baseline and after brushing. To analyze the effect of the type of toothbrush on the plaque, a mixed-effects linear model was used to account for the repeated measurements that yield period, sequence, intrapatient, and interpatient variability.

The difference in scores (baseline minus post-brushing) as average scores were calculated for each participant, for each period, and analyzed for treatment group differences using a mixed model analysis for repeated measures. The factors in the model were subject (random effect), period, treatment, carryover, and baseline whole-mouth average score as the covariate. If the carryover was not significant at the 10% level, it was deleted from the final statistical model. The ICC measured the degree of consistency or reliability between the two examiners for the average pre-brushing and post-brushing scores.

NCSS software® (version 20) was used for data entry and analysis [33]. P-values of <0.05 were considered significant.

## Results

Participant flow

The 24 participants enrolled in the trial were randomized to one of the three treatment sequences. In total, 27 participants were included in the study. Three participants refused to comply with the visits (Figure 1).

Baseline data

The patient characteristics and baseline plaque scores are summarized in Table 2. The mean age was 19.58 ± 1.55 years, with a range of 18-23 years. The majority (n = 13, 54.17%) of the participants were female (Table 2).

**Table 2:** Demographic characteristics of the sample. SD: standard deviation

Characteristics	Mean ± SD	Minimum	Maximum
Age	19.58 ± 1.55	18	23
Gender	N (%)		
Female	13 (54.17)		
Male	11 (45.83)		

Outcome, estimation, precision, and subgroup analyses

The F-test for the treatment was nearly significant (F-value = 14.831, prob level = <0.001) at the 0.05 level. There appeared to be no period effect (F-value = 2.158, prob level = 0.128) and no carryover effect. The treatment differences were statistically significant between the C-TB and Pulsar brush groups (p-value <0.001) favoring the C-TB over the Pulsar brush and between the O-TB and cross-action (p = 0.004) toothbrush favoring the O-TB over the Pulsar brush. However, the difference between the O-TB and C-TB types was not statistically significant (p = 0.063) (Table 3).

**Table 3:** Mixed model analysis.

Treatment	B (SE of B)	β (95% confidence limits)	P-value
Conventional flat trim	0.598 (0.1103)	0.3765–0.8213	<0.001
Orthodontic	0.3339 (0.1095)	0.1132–0.5547	0.0039
Pulsar	Ref		
Individual comparison hypothesis test results			
	Comparison (mean difference)	F-value	Adjusted p-value
Conventional flat trim vs. orthodontic	0.26	5.72	0.063
Conventional flat trim vs. Pulsar	0.59	29.47	<0.001
Orthodontic vs. Pulsar	0.34	9.3	0.011

**Figure 3:**
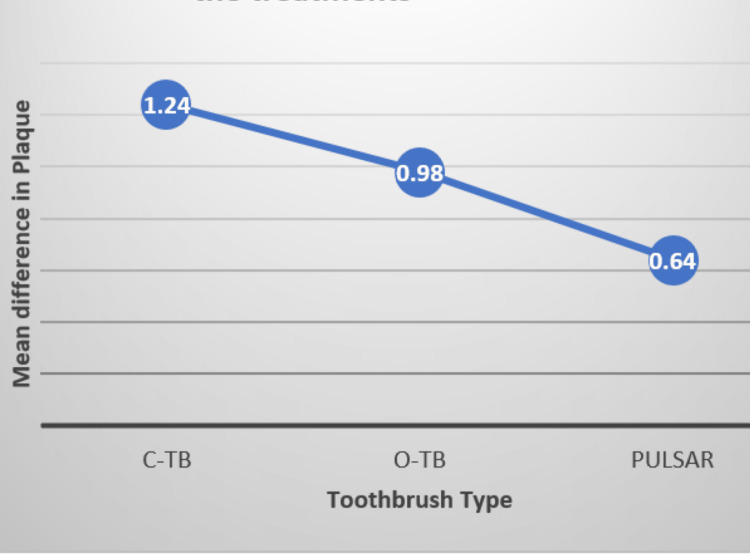
Plot comparing the differences in the means of the plaque scores before and after a single brushing exercise in the three treatment groups. C-TB: conventional flat trim toothbrush; O-TB: orthodontic toothbrush

Figure 3 shows the differences in the means of the plaque scores for the fixed effect (toothbrush type) of the model. A general trend can be seen for the differences in the means of the plaque scores before and after a single brushing exercise in the three treatment groups. A greater plaque reduction in terms of mean difference was seen for the C-TB compared to the O-TB (Figure 3). The lowest difference was seen for the Pulsar-type toothbrush.

The ICC analysis showed a high level of agreement between the two examiners, pre-brushing (ICC ≥ 0.988; p < 0.001) and post-brushing (ICC ≥ 0.973; p < 0.001). These results showed a high degree of consistency or reliability between the two examiners. No serious AEs were observed.

## Discussion

This study investigated the effects of three different manual toothbrushes (C-TB, O-TB, and the battery-operated Pulsar brush) on plaque removal when using the modified Bass method of brushing in orthodontic patients. A significant effect in plaque score was found favoring both the flat trim C-TB and the O-TB compared to the Pulsar toothbrushes. Surprisingly, the difference in plaque score was not significant between the O-TB and C-TB in patients with fixed orthodontic appliances. However, a greater plaque reduction in terms of mean difference was seen for the C-TB compared to the O-TB (Figure 3).

One of the several tools that have been developed for proper oral hygiene maintenance in orthodontic patients includes the specially designed orthodontic manual toothbrush with a unique design of V-shaped grooves along its bristles on the long axis of the toothbrush head. The V-groove nylon bristles gradually shorten, increasing the contact area of ​​the central bracket area, and the long, soft filaments function around the area of the bracket wing. This improves the contact surface between the toothbrush bristles and the orthodontic appliance and improves the hygiene of the patient wearing the fixed orthodontic appliance [15]. Conflicting reports on the effectiveness of the O-TB exist in the literature. Some studies have reported no significant difference between conventional and orthodontic toothbrushes [26,28,30,34]. One study demonstrated a clinically significant reduction in plaque with the orthodontic toothbrush [29]. Another study reported a significant difference in the intergroup comparison of the baseline and follow-up plaque index for the orthodontic toothbrush [30]. In some studies, the improvement in the plaque reduction of the orthodontic toothbrush compared to conventional toothbrushes was not statistically relevant [26,35]; however, there was no report of worsening oral health during re-evaluations.

According to a recent meta-analysis, gingival bleeding is not modified by the O-TB, but major plaque removal of the O-TB by 1.72 (95% confidence interval [CI] = 0.83-2.61; p = 0.0001) was validated [22]. However, additional randomized clinical trials are warranted to clarify the real clinical orthodontic relevance of the orthodontic toothbrush due to the considerable heterogeneity and quality of the data [22]. Our study found no significant difference between the C-TB and O-TB. However, when comparing the mean difference, the C-TB outperformed the O-TB (Figure 3).

On the other hand, studies have reported clinically significant plaque reductions with the Pulsar brush on intact teeth [19-21]. Some studies have compared the Pulsar toothbrush with the powered brushes and did not find a statistically significant difference between the two brushes [35]. The better performance has been attributed to the special features of this brush, as well as the pressure-sensitive split head that conforms to the contours of the teeth to clean the difficult areas and moderately hard surfaces and maintains a moderate amount of pressure on the teeth and gums. In addition, the brush head features innovative bristles (MicroPulse^TM^) that pivot independently (designed to penetrate deep between teeth) and a battery-powered gentle pulse action (lifting food particles and plaque). However, Our findings contradict the findings of previous studies [19-21] in that the Pulsar brush did not perform better than the O-TB or the C-TB (p > 0.05). A possible explanation for this difference could be the additional plaque retention effect of the fixed appliances. This may negate the benefits of brushing with a Pulsar toothbrush in non-orthodontic patients, as cleaning smooth surfaces is more effective.

Study strengths

A key strength of our study is its single-use, three-period, crossover design evaluating whole-mouth plaque removal and treatment sequences including washout phases between study periods. This is an ideal model for the estimation of the period, carryover, and treatment effects. The use of a crossover design has several advantages, including a smaller number of participants required for the study and confounding factors within the participants, such as age, gender, and hand skills. In addition, the effects are measured in the same person. Another strength is that we used two examiners compared with the literature reporting only one examiner. In addition, our study was a short-term, single-use trial. The short-term nature of trials is useful for controlling confounding variables, for example, participant compliance. We also standardized our methodology by adhering to the modified Bass technique, which is recommended by many orthodontists for manual brushes [36]. Two minutes were standardized for brushing the teeth for all three procedures, as well as hygiene instructions were standardized. Some studies followed their own instructions [27,30]. Scrubbing forward and backward was used in another study [29], and modified Bass was used in others [26,28,36]. Additionally, we used conventional fluoride toothpaste without additives that would modify plaque accumulation to prevent the impact of these products on any of the groups.

Study limitations

A major limitation of the study was that the treatments tested were performed as a single exercise; hence, long-term effects could not be assessed. It was not possible to report any AEs associated with prolonged toothbrush use. Well-designed randomized controlled trials are needed to compare the short and long-term effects of different manual toothbrushes. A slightly <90% power in the analyses could be a reason why we did not observe significant differences between the C-TB and the O-TB groups, although the mean difference for the C-TB was much higher compared to the O-TB (Figure 3). Although our study found the C-TB and O-TB superior in the control of plaque, the findings did not assess the gingival parameters. To see an effect on gingival health at least a four-week intervention is required [37]. Even though the patients were not shown the product packaging or provided with additional information about the toothbrushes being tested, they could easily discern the differences between them. Considering this, it is difficult to determine whether the statistically significant difference in plaque reduction was a result of the toothbrushes alone or were due to a placebo effect.

The results of this cross-over randomized controlled trial demonstrated that the Pulsar toothbrushes are not as effective as the C-TB and O-TB in removing dental plaque in participants with fixed orthodontic appliances. This indicates that even though the Pulsar designs appear to work better in intact teeth [20,21], individual factors such as braces can affect the efficacy of a particular design. The level of evidence for the efficacy of the Pulsar toothbrush in orthodontic patients is minimal.

## Conclusions

Within the limitations of this study, the C-TB and O-TB removed significantly more plaque after a single brushing than the Pulsar toothbrush. Nevertheless, the C-TB tested in this study was more effective in removing dental plaque than the O-TB in patients wearing fixed orthodontic appliances. Considering the limitations of this study, additional research is required before evidence-based advice concerning the relative performance of the Pulsar toothbrushes in fixed orthodontic patients can be proven.
